# Digging Deeper to Save the Old Anti-tuberculosis Target: D-Alanine–D-Alanine Ligase With a Novel Inhibitor, IMB-0283

**DOI:** 10.3389/fmicb.2019.03017

**Published:** 2020-01-15

**Authors:** Jianzhou Meng, Peng Gao, Xiao Wang, Yan Guan, Yishuang Liu, Chunling Xiao

**Affiliations:** ^1^Institute of Medicinal Biotechnology, Chinese Academy of Medical Sciences and Peking Union Medical College, Beijing, China; ^2^Department of Microbiology, Li Ka Shing Faculty of Medicine, The University of Hong Kong, Hong Kong, China

**Keywords:** *Mycobacterium tuberculosis*, drug-resistance, D-alanine–D-alanine ligase, inhibitor, D-cycloserine

## Abstract

The emergence of drug-resistant *Mycobacterium tuberculosis* (Mtb) has hampered treatments for tuberculosis, which consequently now require novel agents to overcome such drug resistance. The genetically stable D-alanine–D-alanine ligase A (DdlA) has been deemed as an excellent therapeutic target for tuberculosis. In the present study, a competitive inhibitor (IMB-0283) of DdlA was obtained via high-throughput screening. The minimum inhibitory concentrations (MIC) of IMB-0283 for the standard and clinical drug-resistant Mtb strains ranged from 0.25 to 4.00 μg/mL, whereas the conventional inhibitor of DdlA, D-cycloserine (DCS), only inhibited the growth of the standard Mtb strain at 16 μg/mL. The lethal effect of IMB-0283 on Mtb was found to act intracellularly in a DdlA-dependent manner. Specifically, IMB-0283 prevented the synthesis of neonatal cell walls but did not damage mature cell walls. Compared with those of DCS, IMB-0283 exhibited lower cytotoxicity and a higher selective index (SI). At the same dosages of treatment, IMB-0283 reduced bacterial load (log CFU/mL) in an acute animal model from 5.58 to 4.40, while DCS did not yield any such treatment efficacy. Taken together, the lower cytotoxicity and more efficacious *in vivo* activity of IMB-0283 suggest that it is a promising lead compound for antituberculosis drug development.

## Introduction

Tuberculosis (TB) is remains as a high-burden disease and has claimed millions of lives all over the world due to infections of Mtb. Multidrug-resistant TB occurs in 3.5% of new cases and 18% of previously treated cases, and extensive drug-resistant TB has been on the rise in recent years (WHO, 2018). Hence, conventional chemotherapeutics have become inert in attempts to combat drug-resistant Mtb infections, which consequently now require novel agents to overcome such drug resistance.

Validating whether a therapeutic target with a novel mechanism is worthy of further drug discovery and development is difficult, time-consuming, and requires tremendous resources. To efficiently utilize limited resources, we aimed to leverage a known druggable target since several old drug targets have been previously recognized as promising candidates. Our selection criteria for such old druggable targets were as follows: (1) genetic stability of the target, which may lessen the probability of drug resistance; and (2) the present inhibitor of the target is restricted due to severe side effects but not drug resistance. D-alanine–D-alanine ligase A (DdlA, EC 6.3.2.4, and Rv2981c), the target of DCS (Halouska et al., 2014), is an excellent drug target for treating TB since its mutation rate to generate resistant strains is much smaller than that of other targets (McGrath et al., 2014). However, the neurological and psychiatric side effects of DCS limit its clinical application (Kass and Shandera, 2010). Therefore, it is necessary to exploit novel anti-TB compounds that target Mtb DdlA. In the present study, a safe and low-toxicity inhibitor of DdlA was obtained with potent anti-TB activity both *in vitro* and *in vivo*.

## Materials and Methods

### Bacteria and Plasmids

All chemicals used in this study were purchased from Sigma (Sigma-Aldrich, St. Louis, MO, United States) unless otherwise stated. Mtb H37Rv (ATCC27294) and other clinical drug-resistant strains conserved by the Chinese Center For Disease Control And Prevention were cultured in Middlebrook 7H9 broth (supplemented with glycerol and polysorbate 80) in combination with Middlebrook albumin-dextrose-catalase (ADC) enrichment or 7H10 agar solid media supplemented with oleic acid-albumin-dextrose-catalase (OADC) enrichment (ADC + 0.003% oleic acid) (Allen, 1998). *Escherichia coli* (*E. coli*) DH5α and *E. coli* BL21 (DE3) plyS (TransGen Biotech, Inc., Beijing, China) were cultured in Luria–Bertani (LB) broth or on LB agar plates. Plasmid pET28a (+) was conserved by our lab and plasmid pAZI9479 was kindly gifted by Professor Francesca Forti (Forti et al., 2009). Kanamycin was added at concentrations of 100 μg/mL for *E. coli*, while hygromycin was added at 200 μg/mL for *Escherichia coli* and at 100 μg/mL for Mtb. Isopropyl β-D-1-thiogalactopyranoside (IPTG) was used as an inducer to express DdlA in *E. coli* BL21 (DE3) plyS, and pristinamycin (Santa Cruz Biotech, Santa Cruz, CA, United States) was used to induce gene expression in Mtb.

### Molecular Manipulations

The Mtb H37Rv genome was extracted from the log phase cells as previously described in Mtb protocols (Gordhan and Parish, 2001). All PCR reagents were purchased from TransGen Biotech. The primers for amplification *ddl*A were designed using software primer 5.0 based on the sequence in the NCBI database (GenBank accession number: 888415). The primers *ddl*A F (5’-AAAAGAATTCGTGAGTGCTAACGACCGGC-3′) and *ddl*A R (5′-AAAAAAGCTTCTAGTGCAGGCCCACGCCG-3′) were used to amplify the whole fragment of *ddl*A for protein expression, and primers *ddl*AM F (5′-AATTCCATGGGTGAGTGCTAACGACCGGC-3′) and *ddl*AM R (5′-CGCCCATATGGGGTTTGACGAACACCGGTA-3′) were used to synthesize the former fragment of *ddl*A for constructing a conditional mutant strain. The lineated parts of the sequences denote the limited digestion sites (*Eco*RI, *Hin*dIII, *Nco*I, and *Nde*I).

The purified PCR product of the *ddl*A gene was cloned into plasmid pET28a (+)*Eco*RI-*Hin*dIII sites to generate pET28a (+)-*ddl*A; the fragment of *ddl*AM was cloned into pAZI9479 *Nco*I-*Nde*I sites to obtain pAZI9479-DM. Sequencing was subsequently performed to ensure that there were no mutations.

### Construction of the Conditional Mutant Bacteria

The bacteria and plasmids were treated as previously described (Meng et al., 2015). First, 1 μg of UV-illuminated plasmid pAZI9479-DM (no more than 5 μL) was mixed with 200 μL of competent cells in a 0.2-cm electroporation cuvette (Bio-rad, Hercules, CA, United States), and the cuvette was pulsed at a strength of 2.5 kV, 25 uF, and 1000 Ω resistance with a Gene Pulser Xcell (Bio-rad). These bacteria were resuscitated in 5 mL of 7H9 broth for 24 h at 37°C and were then spread on 7H10 plates containing 0.5 μg/mL of pristinamycin and 100 μg/mL of hygromycin that were incubated at 37°C for 4 weeks until colonies formed. Plasmid-positive bacteria were determined via sequencing, and the resultant mutant strain was designated as Mtb-KD.

### Expression and Purification of DdlA

The log-phase *E. coli* BL21 (DE3) pLysS-bearing plasmid pET28a (+)-*ddl*A were induced by IPTG (final concentration of 0.3 mM) at 28°C for 8 h, and the recombinant protein carrying a hexahistidine tag at the N-terminal was purified by Ni^2+^ ion-affinity chromatography using a bouncing gradient of 40–100–200–400 mM imidazole in washing buffer (20 Mm of Tris–HCl, pH of 8.0, 500 mM of NaCl, 1 mM of dithiothreitol). The eluted fractions were analyzed by SDS-PAGE and were visualized with Coomassie Brilliant Blue R-250 gel staining. The purified DdlA was desalted on a PD-10 column (GE Healthcare, Piscataway, NJ, United States), concentrated through a 10-kDa cut-off Millipore Centricon device (Millipore, Billerica, MA, United States), and was stored at −80°C with 50% glycerol. The concentration of the enzyme was identified via the BCA protein assay kit (TransGen Biotech).

### Kinetic Analysis of DdlA

The activity of DdlA was monitored by coupling with pyruvate kinase (PK) and lactate dehydrogenase (LDH; Sigma-Aldrich) to detect the release of ADP. Reactions were proceeded in 96-well plates (at 37°C) containing 50 mM of Tris–HCl (pH 8.0), 10 mM of MgCl_2_, 10 mM of KCl, 1 mM of dithiothreitol, 250 μM of ATP, 1000 μM of D-alanine (D-Ala), 1 mM of phosphoenolpyruvic acid (PEP), 0.5 mM of NADH, 2 μg of DdlA, 1 U of pyruvate kinase (PK) and 1 U of lactate dehydrogenase (LDH). The assay system was monitored via a Perkin Elmer EnSpire^®^ 2300 Multimode Plate Reader (PerkinElmer, MA, United States) by detecting decreases in NADH.

The calibration curve of the ultraviolet absorption of NADH at 340 nm to its concentrations was fabricated before studying the enzymatic kinetics of DdlA. The kinetic parameters of DldA were determined using serial twofold dilutions of one substrate (from 1 mM to 31.25 μM), while the other substrate was held constant at 1 mM. Km and Vmax values were calculated using non-linear regression of the Michaelis-Menten model in GraphPad Prism 5.0 (GraphPad Software, Inc., San Diego, CA, United States). Kcat was calculated based on the molecular weight of DdlA (42 kDa).

### Screening of Inhibitors

A high-throughput screening assay was designed according to kinetic parameters. Systems containing 1 μL of dimethyl sulfoxide (DMSO) were used as negative controls, while the positive control systems contained heat-inactivated DdlA. Specifically, 1 μL of samples diluted at 2 mg/mL were added to a 96-well plate with a final concentration of 20 μg/mL. Parameters signal window, Z′ factor, and an assay variability ratio were used to assess the reliability of the model (Iversen et al., 2006). Via this model, we screened through 150,000 synthetic compounds. The inhibition rate was calculated as follows:

$$\text{IR}=\left(1-\frac{\text{A}\,p-\text{A}\,s}{\text{A}\,p-\text{A}\,n}\right)\times 100\%$$

in which the IR, A*n*, A*s*, and A*p* denote the inhibition rate and the ultraviolet absorption of the negative control, sample, and positive control, respectively. The IR threshold was defined at 30%. The IRs of inhibitors in twofold serial dilutions to DdlA were detected to calculate their IC_50_ values using the non-linear regression module of GraphPad Prism 5.0. The reaction rates of systems containing various concentrations of substrate (100, 200, 300, 400, 500, and 600 μM) and inhibitors (10 or 40 μg/mL) were detected to determine their inhibitory modes. Lineweaver-Burk plots and Dixon plots were applied to analyze the results.

### Antibacterial Activity *in vitro*

The minimal inhibitory concentration (MIC) of IMB-0283 (J&K Chemical Company, Beijing, China, synthesized by Enamine) to Mtb was determined as previously described (Darby and Nathan, 2010). Briefly, the mid-log phase H37Rv and the other clinical drug-resistant strains were ultrasonically suspended and adjusted to a final concentration of 1–2 × 10^5^ CFU/mL in 7H9 broth. Then, 100 μL of suspension was exposed to the compound in serial twofold dilutions, from 128 μg/mL to 0.063 μg/mL, in 96-well plates in triplicate. INH and RMP were used as positive controls. After 2 weeks of incubation at 37°C, the viability of bacteria was detected using a resazurin microtiter assay (Montoro et al., 2005). The synergistic effects of IMB-0283 with INH and RMP were tested as previously described (Shen et al., 2010). INH and RMP were twofold diluted from 16 μg/mL to 0.008 μg/mL, and their MIC values to H37Rv were determined when the concentration of IMB-0283 was set at 0.25 × MIC.

The sensitivity of Mtb-KD cultured with higher or lower concentrations of pristinamycin (0.1 and 0.1 × 10^–4^ μg/mL) to IMB-0283 was tested to confirm if it interacted with DdlA in bacteria, and DCS was used as a positive control.

### Morphology of Mtb Treated With IMB-0283

The log phase of Mtb H37Rv was treated with 0.35 μg/mL of IMB-0283 and was incubated for 12 h or 7 days, and Mtb morphological transformations were detected as previously described (Shen et al., 2010). The washed bacterial pellets were spotted on Si chips (Ted Pella, Inc., Redding, CA, United States) and were fixed with 2.5% glutaraldehyde. The chips were washed and dehydrated in a graded ethanol series. Samples were coated with Au/Pd (E-1045 ion sputter coater, Hitachi High Technologies Co., Tokyo, Japan) after being critical point-dried (Bal-Tec CPD 030 Critical Point Dryer, Bal-Tec AG, Liechtenstein, Germany). The bacteria morphologies were surveyed by a Quanta200 scanning electron microscope (FEI, Oregon, United States).

### Cytotoxicity Assay

HepG2 cells (*ATCCHB-8065*) and Vero green-monkey kidney cells (*ATCCC1008*) were cultured in Dulbecco’s modified eagle’s medium (DMEM) supplemented with 10% fetal bovine serum (FBS) and 1% antibiotics (100 U/mL penicillin), and were incubated at 5% CO_2_ in a humidified atmosphere at 37°C (Heracell 150, Thermo Electron Corp., Waltham, MA, United States). After 24 h of incubation, the adhered exponential cells (1 × 10^5^ cells/100 μL) were washed three times with fresh medium. IMB-0283 was added at a triple dilution from 900 μg/mL to 1.23 μg/mL with the medium, while DCS was diluted from 3000 μg/mL to 4.11 μg/mL. DMSO (1%) was added to induce a solvent effect, and normal-growth cells were used as negative controls. 3-[4, 5-dimethylthiazol-2-yl]-2, 5-diphenyl tetrazolium bromide (MTT) were used to detect cell viability at 2 days later (Correia et al., 2014). TC_50_ values were calculated using non-linear regression (curve fit) of the log inhibitor vs. the normalized response-variable slope module of GraphPad Prism 5.0.

### IMB-0283 Activity in a Mouse Model of TB

Specific pathogen-free (SPF) male Balb/c mice were purchased from the Institute of Laboratory Animal Sciences at the Chinese Academy of Medical Sciences and Peking Union Medical College. All animal experiments were supervised and approved by the Institutional Animal Care and Use Committee of the Institute of Medicinal Biotechnology. Twenty-six mice (6–8 weeks old, 18–20 g) were infected with Mtb H37Rv (about 100 CFU) via the 099C A4224 Inhalation Exposure System (Glas-col, Terre Haute, IN, United States). At 15 days after inoculum, two mice were anesthetized and decapitated to confirm the success of infection. DCS, INH, IMB-0283, and vehicle (0.5% CMC-Na) were administered through a gavage route (six mice per group). All chemicals were dosed at 25 mg/kg. Mice were sacrificed after 15 administrations (five times per week) to quantify pulmonary bacterial loads via counting CFUs.

## Results

### Expression, Purification, and Kinetic Characterization of DdlA

**FIGURE 1:**
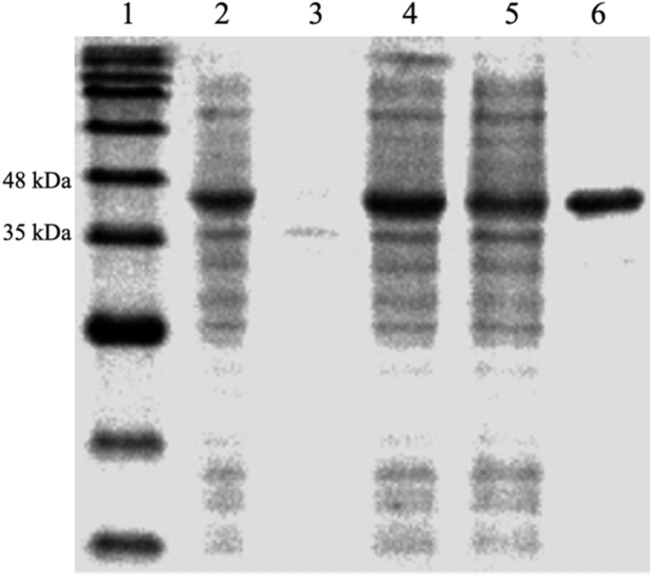
SDS-PAGE analysis of the expression and purification of enzyme DdlA. Lane 1 represents the pre-sharp protein standard. Lane 2 shows the whole protein of the recombinant cell. Lane 3 shows the precipitate protein. Lane 4 shows the supernatant fluid. Lane 5 shows the effluent liquid. Lane 6 shows the purified DdlA.

The protein, DdlA, was purified from *E. coli* BL21 (DE3) pLysS-harboring pET28a (+)-ddlA via immobilized metal affinity chromatography with Ni^2+^-NTA agarose. The purified protein was verified by SDS-PAGE (Figure 1), and DdlA (42 kDa) was visualized between 35 and 48 kDa.

**FIGURE 2:**
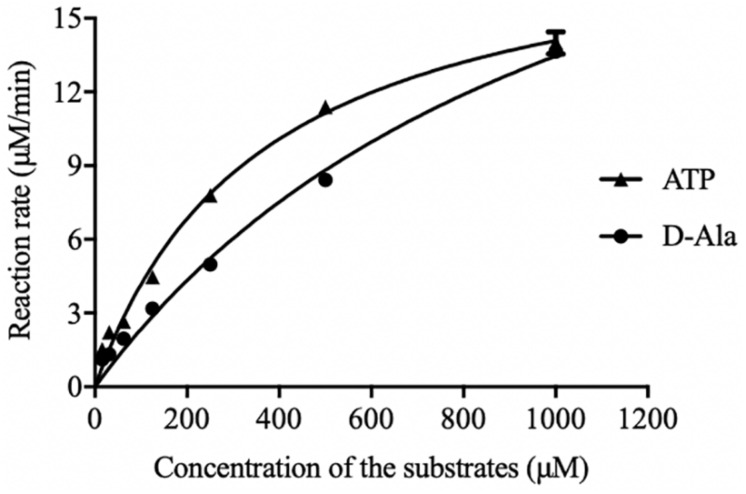
Initial reaction rates of DdlA affected by concentration of ATP and D-Ala. The kinetics parameters of DdlA were calculated via the Michaelis-Menten model. The reaction rates affected by ATP and D-Ala (which were twofold diluted from 1 mM to 31.25 μM) were detected to calculate the kinetic parameters via non-linear regression of the Michaelis-Menten model in GraphPad Prism 5.0 (*n* = 3).

The initial reaction rate of DdlA was determined by correlating the concentrations of ATP and D-Ala to the UV absorbances of NADH. The reaction rates of DdlA affected by concentrations of substrates were detected to calculate its enzymatic kinetic parameters (Figure 2 and Table 1). The Kms for ATP and D-Ala were 450.6 μM and 1780 μM, respectively, and their Vmax values were 20.29 μMmin^–1^ and 36.61 μMmin^–1^, respectively. Kcat values, calculated by dividing Vmax with the enzyme concentration, for ATP and D-Ala were 405.8 min^–1^ and 732.19 min^–1^, respectively. These results were consistent with those reported previously (Prosser and de Carvalho, 2013).

**TABLE 1:** Kinetic parameters of DdlA.

**Substrate**	**Km (μM)**	**Vmax (μMmin^–1^)**	**Kcat^a^ (min^–1^)**
ATP	359.90 ± 30.53	19.15 ± 0.705	383.00 ± 14.14
D-Ala	1113.00 ± 153.90	28.44 ± 2.444	568.79 ± 48.93

*Kcat^*a*^ was calculated via Vmax/[E].*

### HTS Assay and Identification of DdlA Inhibitor

**FIGURE 3:**
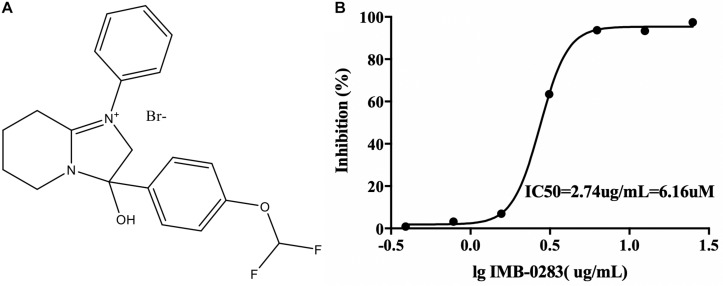
The structure of IMB-0283 and its IC_50_ value for DdlA. **(A)** The structure of IMB-0283. **(B)** The IC_50_ value of IMB-0283 to DdlA. Results are presented as the mean ± SD (*n* = 3).

The A high-throughput screening assay was established according to the enzymatic parameters of DdlA. The signal window, Z′-factor and assay variability ratio of the screening model were 12.87, 0.76, and 0.24, respectively, indicating that it was a reliable screening model. Via this model, 48 compounds (inhibition ratio ≥ 30%) were obtained from our library (the positive rate was 0.032%). Among these inhibitors, IMB-0283 displayed the most potent inhibitory activity to DdlA, with an IC_50_ of 6.16 μM (Figure 3).

**FIGURE 4:**
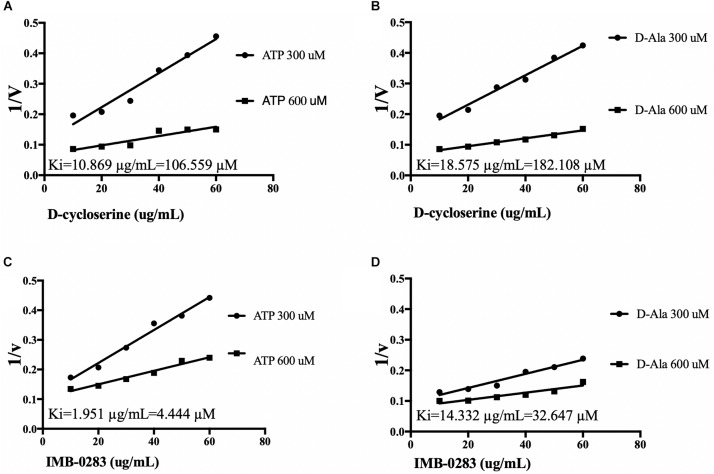
Ki values of inhibitors calculated by Dixon-plots for DdlA. Reactions were monitored when inhibitors varied from 10 to 60 μg/mL, while one substrate was fixed at 1000 μM and the other substrate was either 300 or 600 μM (*n* = 3). Panels **(A,B)** show the Ki values of DCS to DdlA. Panels **(C,D)** show the Ki values of IMB-0283 to the enzyme.

The reaction rates of the assays affected by various concentrations of inhibitors and substrates were measured to analyze inhibitory modes and to calculate Ki values. IMB-0283 competed with both substrates of DdlA with Ki values of 4.444 μM (ATP) and 32.647 μM (D-Ala) (Figure 4). Ki values of DCS for these substrates were 106.599 μM and 182.108 μM, indicating that IMB-0283 had a better affinity to DdlA compared to that of DCS.

### Anti-tuberculosis Activity of IMB-0283

IMB-0283 potently inhibited H37Rv at an MIC of 0.5 μg/mL, while the MIC of DCS was 16 μg/mL. IMB-0283 also displayed antibacterial activity to several clinical strains, with MICs ranging from 0.5 μg/mL to 4.0 μg/mL (Table 2), while DCS did not show any inhibitory effect. These results indicated that IMB-0283 had no cross-resistance with that of conventional anti-TB drugs.

**TABLE 2:** Anti-tuberculous activity of IMB-0283.

**Mtb strains**	**Drug sensitivity background^∗^**							**MIC (μg/mL)**
		
	**INH**	**RMP**	**EMB**	**STR**	**CPM**	**KAN**	**OFX**	
H37RV	S	S	S	S	S	S	S	0.5
FJ05349	S	S	S	S	S	S	S	0.5
FJ05060	S	S	S	S	S	S	S	0.5
FJ05195	R	R	R	R	S	R	R	4.0
FJ05120	R	R	S	S	S	S	S	2.0
FJ05189	R	R	S	S	S	S	S	1.0
xz	R	R	S	R	R	S	R	4.0

*INH, isoniazid; RMP, rifampin; EMB, ethambutol; STR, streptomycin; CPM, cefpiramide; KAN, kanamycin; OFX, ofloxacin; MIC, minimum inhibitory concentrations; S, sensitive; R, resistant.*

The MIC of INH against H37Rv was reduced eightfold, from 0.125 μg/mL to 0.016 μg/mL, when combined with IMB-0283 at 0.125 μg/mL. For RMP, the MIC decreased from 0.063 μg/mL to 0.016 μg/mL. This finding suggests that there is a synergistic effect between IMB-0283 and either INH or RMP.

### IMB-0283 Intracellularly Interacts With DdlA

**FIGURE 5:**
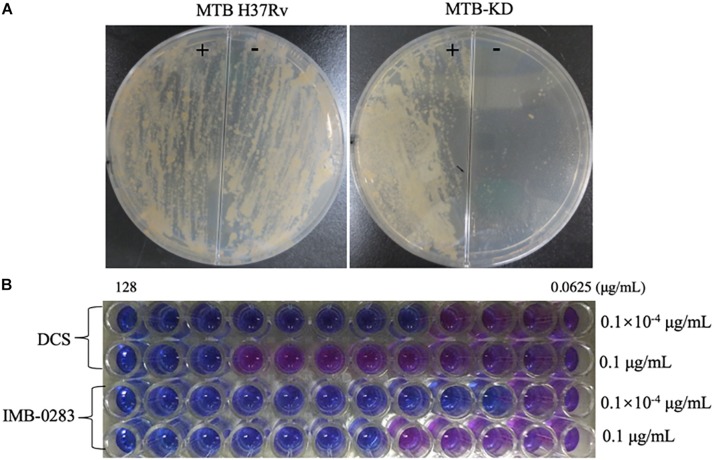
DdlA is essential for Mtb growth and IMB-0283 inhibits Mtb growth in a DdlA-dependent manner. A conditional mutant strain, Mtb-KD, and its sensitivities to DCS and IMB-0283 were confirmed by culturing with different concentrations of pristinamycin. **(A)** Wild-type and mutant Mtb H37Rv were lineated on 7H10 solid medium in the presence (+) or absence (−) of 0.1 μg/mL of pristinamycin; the former formed lawns on the medium independent of the presence of the inducer, while the latter formed colonies only on the medium containing inducer. **(B)** DCS inhibited mutant Mtb at 1 μg/mL or 32 μg/mL when cultured with 0.1 × 10^– 4^ μg/mL (repressed) or 0.1 μg/mL (induced) of inducer. At the same conditions, IMB-0283 inhibited the mutant strain at either 0.25 or 2 μg/mL.

A fragment (about 600 bp) of *ddl*A starting from ATG was cloned into pAZI9479 to generate pAZI9479KD. The mutant Mtb-KD was generated by inserting pAZI9479KD into the Mtb chromosome. In this mutant strain, the expression of *ddl*A was under the control of pristinamycin. A higher concentration of pristinamycin (0.1 μg/mL) led to overexpression of *ddlA*, while a lower concentration of pristinamycin (0.1 × 10^–4^ μg/mL) corresponded to knock-down of *ddlA*. Figure 5A shows that the mutant bacteria were not able to grow on the 7H10 plates without pristinamycin, while the growth of wild-type Mtb was independent of pristinamycin. These results demonstrate that DalA was essential for Mtb. Susceptibilities of Mtb-KD cultured with different concentrations of inducer to IMB-0283 were detected to confirm whether the lethal effect was dependent on inhibiting DdlA intracellularly. When the *ddlA* gene was knocked-down, IMB-0283 and DCS inhibited the mutant at 0.25 μg/mL and 1 μg/mL, respectively. Their MIC values climbed to 2 μg/mL and 32 μg/mL, respectively, when the *ddlA* gene was overexpressed (Figure 5B). Consistent with the findings of DCS, the divergences of MIC values indicated that IMB-0283 inhibited Mtb bacteria in a DdlA-dependent manner.

### Morphological Alterations of Mtb Treated With IMB-0283

**FIGURE 6:**
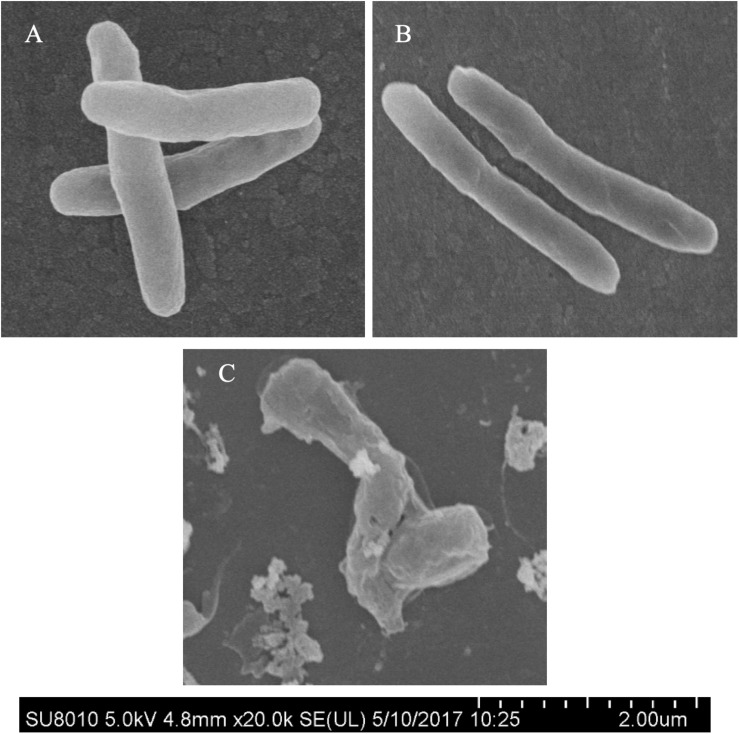
Morphological transformations of Mtb treated with IMB-0283. **(A)** The log phase Mtb. **(B)** Mtb treated with IMB-0283 for 12 h. Bacteria in **(A,B)** were uniform and exhibited a typical long-rod morphology. **(C)** Morphologies of Mtb treated for 7 days became shrunken, shorter, and fragmented.

Morphological changes of Mtb treated with IMB-0283 were monitored to determine whether the inhibitor of DdlA altered the shape of Mtb. Mtb bacteria had a typical long-rod morphology resembling the log phase of bacteria at 12 h after treatment (Figures 6A,B). Shapes of these bacteria changed dramatically by 7 days after exposure to IMB-0283, during which they became shrunken, shorter, and fragmented (Figure 6C). These results indicate that IMB-0283 inhibited and killed Mtb by inhibiting cell-wall synthesis but not destroying mature cell walls.

### Cytotoxicity of IMB-0283

Next, the TC_50_ values of IMB-0283 to HepG2 and Vero cells were evaluated (Table 3). Although the TC_50_ values of DCS for these mammalian cells were larger than 1,000 μg/mL, the SI index of DCS was only about 80. While the TC_50_ values of IMB-0283 for HepG2 and Vero cells were 115.4 μg/mL and 249.8 μg/mL, respectively, the SI index of IMB-0283 was far greater than 200. These results indicated that IMB-0283 might be safer than DCS.

**TABLE 3:** Cytotoxicity profiles of IMB-0283 compared with those of DCS.

**Compound**	**MTBH37Rv**	**HepG2**		**Vero**	
	**MIC (μg/mL)**	**TC_50_ (μg/mL)**	**SI**	**TC_50_ (μg/mL)**	**SI**
DCS	16	1380	86.25	2044	127.75
IMB-0283	0.5	115.4	230.8	249.8	499.6

*DCS, D-cycloserine; MIC, minimum inhibitory concentrations; SI, selective index which was calculated via TC_50_/MIC.*

### IMB-0283 Activity in a Mouse Model of TB

**FIGURE 7:**
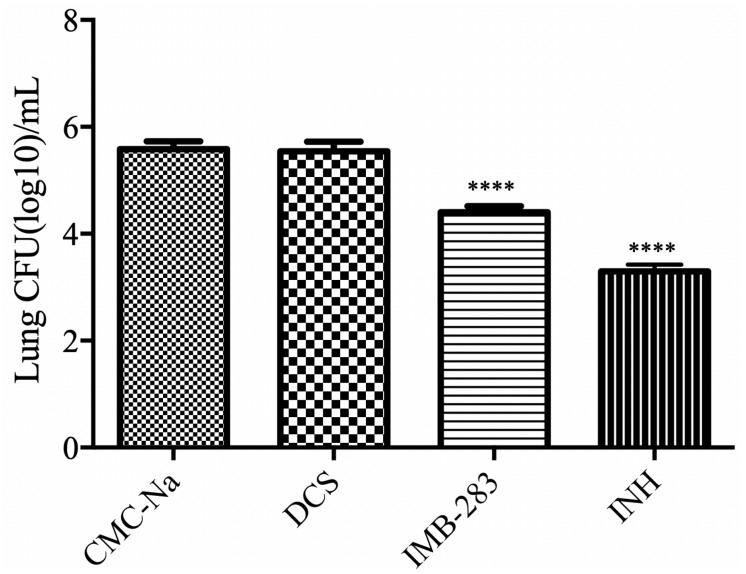
Bacteriostatic activity of IMB-0283 in BALB/C mice. DCS, IMB-0283, and INH were each administered at 25 mg/kg 15 times, and the vehicle CMC-Na was used as a negative control. INH and IMB-0283 reduces the bacterial load in lungs from 5.58 to 3.29 or 4.40 (log CFU/mL), respectively, but DCS showed no antibacterial activity (*P* > 0.5). ^****^Statistically significant difference from the negative control (*P* < 0.0001; *n* = 6).

Finally, the antibacterial activity of IMB-0283 in a mouse model of TB was evaluated (Figure 7). After 15 administrations (five times per week) at a dosage of 25 mg/kg, INH reduced the Mtb load in mouse lungs from 5.58 to 3.29 (log CFU/mL), but DCS did not yield any antibacterial activity (*P* > 0.5). IMB-0283 significantly decreased the bacteria load to 4.40, exhibiting much more potent antibacterial capacity than that of DCS (*P* < 0.0001).

## Discussion

The selection of a specific therapeutic target for high-throughput screening determines the output of lead compounds; hence, it is questionable to establish high-throughput screening models based on essential gene products to search for inhibitors, as there are often considerable discrepancies between the essentiality and drugability of the resultant proteins (Gashaw et al., 2011). Therefore, we aimed to identify and test novel anti-mycobacterial compounds based on previously confirmed drug targets. The integrity of the cell wall is vital in supporting cell growth and virulence, as well as in influencing antimicrobial resistance (Abrahams and Besra, 2018). The processes for synthesizing and assembling cell walls have been targeted by several chemotherapeutics (Bhat et al., 2017). The reticular peptidoglycan of Mtb not only withstands exoteric osmotic pressure, but also provides anchor sites for other cell-wall components (Kieser and Rubin, 2014). The generation of peptidoglycans starts from the synthesis of intracellular glycopeptide precursors that are matured by conjugating with d-alanyl-d-alanine. The synthesis of this dipeptide can be interrupted by DCS, and DdlA has been shown to be the primary target of DCS (Halouska et al., 2014). Although the severe side effects of DCS have limited its application in TB treatment, it has been demonstrated that DdlA is much more stable genetically than other anti-TB drug targets (McGrath et al., 2014). Therefore, researchers have attempted to exploit novel inhibitors that may supersede DCS (Wu et al., 2008; Triola et al., 2009; Vehar et al., 2011; Ameryckx et al., 2018). However, most screening assays have been carried out using DdlB of *E. coli*, the crystal structure of which is distinct from that of DdlA in Mtb (Bruning et al., 2011). Whether or not these inhibitors inhibit the growth of Mtb has not yet been reported. Consequently, there has been a continued need to search for novel inhibitors of Mtb DdlA specifically.

In the present study, a credible high-throughput screening model was established to search for specific inhibitors of Mtb DdlA from our chemical library. The cut-off value was set at 30% to obtain as many compounds as possible and to exclude ineffective inhibitors. Forty-eight compounds were obtained that had considerable inhibitory capacities to the Mtb enzyme. Among these chemicals, IMB-0283 competitively inhibited DdlA similar to that of DCS, and it nearly equally inhibited both standard and clinical drug-resistant Mtb strains. IMB-0283 also exhibited bacteriostatic effects of both INH and RMP *in vitro*. We consulted PubChem for analogs of IMB-0283 for further information. There were 238 similar analogs that were obtained via PubChem, but little information regarding their biological activities were reported. This suggested that IMB-0283 may represent the first of this category to be found to inhibit Mtb. Future studies should test the inhibitory capacities of a series of several derivatives to DdlA of Mtb to analyze the structure-activity relationships of IMB-0283, which may provide clues to further optimize IMB-0283. Through measuring sensitivities of our conditional mutant strain, Mtb-KD, cultured with low or high inducers to IMB-0283, we found that IMB-0283 inhibited the catalytic activity of DdlA intracellularly. Although the larger divergences in enzyme-inhibitory and antibacterial activities suggested that DdlA may not be the primary target of IMB-0283, IMB-0283 can interfere with the formation of cell walls but do not damage mature cell walls. Compared with properties of DCS, IMB-0283 exhibited less cellular toxicity and more bacteriostatic activity in a mouse model of TB.

Although the present study elucidated that IMB-0283 may represent a promising drug for the TB treatment, further research is needed to better understand its underlying mechanisms and to further confirm its low toxicity. In future studies, we plan to investigate the interactions of IMB-0283 with DdlA through crystallization, by which we may be able to optimize IMB-0283 in order to develop more potent and specific inhibitors. Additionally, we plan to screen for resistant Mtb strains to evaluate the probability to generate IMB-0283-resistant mutations, as well as to expound upon mechanisms by which IMB-0283 inhibit Mtb. Lastly, comprehensive studies should be conducted to determine the adsorption, distribution, metabolism, excretion, and toxicity (ADMET) properties of these IMB-0283-modified compounds. Through these future studies, we expected to identify other promising anti-TB agents based on IMB-0283.

## Data Availability Statement

The raw data supporting the conclusions of this article will be made available by the authors, without undue reservation, to any qualified researcher.

## Ethics Statement

The animal study was reviewed and approved by the Institutional Animal Care and Use Committee of the Institute of Medicinal Biotechnology.

## Author Contributions

JM and PG: experimental operation and manuscript preparation. XW and YG: data collection. YL: data analysis. CX: literature search and experimental design.

## Conflict of Interest

The authors declare that the research was conducted in the absence of any commercial or financial relationships that could be construed as a potential conflict of interest.

## Abbreviations

DCS: D-cycloserine
DdlA: D-alanine –D-alanine ligase A
INH: isoniazid
MIC: minimum inhibitory concentrations
Mtb: *Mycobacterium tuberculosis*
RMP: rifampin
SI: selective index
TB: tuberculosis.
